# Mitochondrial Dysfunction and Oxidative Stress in Retinal Degeneration: Mechanisms, Biomarkers, and Therapeutic Perspectives

**DOI:** 10.3390/cimb48060612

**Published:** 2026-06-11

**Authors:** Feliciana Menna, Stefano Lupo, Laura De Luca, Antonio Baldascino, Enzo Maria Vingolo, Alessandro Meduri

**Affiliations:** 1 Department of Medical-Surgical Sciences and Biotechnologies, U.O.C. Ophthalmology, Sapienza University of Rome, Via Firenze 1, 04019 Terracina, Italy; 2 Department of Biomedical and Dental Science and of Morphological and Functional Images, University of Messina, 98122 Messina, Italy; 3 Ophthalmology Unit, Fondazione Policlinico Universitario A. Gemelli IRCCS, 00168 Rome, Italy

**Keywords:** retinal degeneration, mitochondrial dysfunction, oxidative stress, reactive oxygen species, mitophagy, retinal pigment epithelium, biomarkers, mitochondrial therapy

## Abstract

Mitochondrial dysfunction and oxidative stress are increasingly recognized as key contributors to the development and progression of retinal degenerative diseases, including age-related macular degeneration and inherited retinal dystrophies. Growing evidence suggests that alterations in mitochondrial function, excessive production of reactive oxygen species, defective mitophagy, and chronic inflammatory responses are closely interconnected processes that contribute to retinal cell damage and degeneration. This review provides an overview of the current understanding of the molecular mechanisms linking mitochondrial dysfunction to retinal degeneration, with particular emphasis on the impact of oxidative stress, mitochondrial quality-control pathways, and inflammatory signaling. Available evidence indicates that mitochondrial DNA damage, impaired bioenergetics, and dysregulated mitochondrial dynamics play a crucial role in the degeneration of photoreceptors and retinal pigment epithelium cells. In turn, oxidative stress further exacerbates mitochondrial impairment, creating a self-sustaining cycle that promotes disease progression. Recent advances have also highlighted the therapeutic potential of targeting mitochondrial pathways. Although several mitochondria-directed strategies have shown encouraging results in experimental models, their translation into clinical practice remains at an early stage. Overall, the available data identify mitochondria as a promising therapeutic target and support the development of precision medicine approaches aimed at preserving retinal function and slowing disease progression in patients with retinal degenerative disorders.

## 1. Introduction

Retinal degenerative diseases are among the leading causes of irreversible vision loss worldwide and include a broad spectrum of conditions, ranging from inherited disorders such as retinitis pigmentosa to complex age-related diseases like age-related macular degeneration (AMD). Although remarkable progress has been made in recent years in genetic diagnostics, retinal imaging, and the understanding of disease mechanisms, therapeutic options capable of halting or reversing disease progression remain limited. Increasing evidence suggests that mitochondrial dysfunction and oxidative stress are not merely secondary consequences of retinal damage but may represent key events in the early stages of degeneration, contributing directly to disease onset and progression [1,2].

The retina has one of the highest metabolic demands of any tissue in the human body. Photoreceptors and retinal pigment epithelium (RPE) cells require a continuous supply of energy to support phototransduction, ion transport, and synaptic activity. To meet these demands, retinal cells rely heavily on mitochondrial oxidative phosphorylation, making them particularly vulnerable to mitochondrial impairment [3]. At the same time, the retina operates in an environment characterized by intense oxygen consumption and constant exposure to visible light, conditions that favor the generation of reactive oxygen species (ROS) and increase susceptibility to oxidative damage [4].

Growing experimental and clinical evidence indicates that mitochondrial alterations occur early in retinal degenerative diseases, often before significant structural damage becomes evident. Impaired mitochondrial bioenergetics, excessive ROS production, defective mitophagy, and dysregulated inflammatory responses appear to interact in a complex network that ultimately promotes photoreceptor dysfunction and cell death. Rather than acting as isolated mechanisms, these processes reinforce one another, creating a self-perpetuating cycle of cellular stress and degeneration.

A better understanding of the relationship between mitochondrial dysfunction, oxidative stress, and inflammation may provide new opportunities for early diagnosis and therapeutic intervention. In this context, the present review summarizes current evidence regarding the molecular mechanisms linking mitochondrial impairment to retinal degeneration, discusses emerging biomarkers of mitochondrial dysfunction, and evaluates the potential of mitochondria-targeted therapeutic strategies as novel approaches for preserving retinal function and preventing vision loss.

## 2. Mitochondrial Structural and Functional Alterations

Mitochondrial dysfunction is increasingly recognized as one of the earliest and most consistent features of retinal degeneration. Structural studies have revealed profound mitochondrial alterations in both photoreceptors and RPE cells, including mitochondrial swelling, fragmentation, and disruption of cristae organization [5,6]. These morphological abnormalities reflect a loss of mitochondrial integrity and are closely linked to impaired cellular energy metabolism.

One of the main consequences of mitochondrial impairment is dysfunction of the electron transport chain, which reduces ATP production while increasing electron leakage and the subsequent generation of ROS [7]. Given their exceptionally high energy requirements, photoreceptors are particularly vulnerable to this bioenergetic deficit. As mitochondrial efficiency declines, retinal cells become less capable of meeting their metabolic demands, ultimately compromising visual function.

In parallel, accumulating evidence indicates that mitochondrial DNA (mtDNA) damage—including mutations, deletions, and oxidative modifications—further contributes to mitochondrial dysfunction. Such alterations have been identified in both aging retinas and experimental models of retinal disease, suggesting a common mechanism underlying retinal vulnerability [8,9].

Beyond structural and genetic changes, recent studies have highlighted disturbances in mitochondrial biogenesis. Reduced activity of key regulators, including peroxisome proliferator-activated receptor gamma coactivator-1 alpha (PGC-1α) and nuclear respiratory factors, appears to impair the ability of retinal cells to replace damaged mitochondria and maintain adequate energy production [10]. As a result, the compensatory mechanisms that normally preserve mitochondrial homeostasis become progressively less effective.

Notably, several experimental studies suggest that mitochondrial dysfunction develops before overt photoreceptor degeneration becomes evident, supporting the concept that mitochondrial impairment is not simply a consequence of retinal damage but may act as an early driver of disease progression [11]. Collectively, these findings emphasize the central role of mitochondrial homeostasis in retinal health and provide a strong rationale for targeting mitochondrial pathways in neuroprotective therapeutic strategies.

The principal mitochondrial alterations and their downstream effects are summarized in Table 1.

## 3. Oxidative Stress and ROS-Mediated Damage

Oxidative stress is widely recognized as a key mechanism linking mitochondrial dysfunction to retinal degeneration. Increased levels of reactive oxygen species (ROS) have been consistently reported in both experimental models and patients with retinal diseases, supporting their central role in retinal injury [13]. Under physiological conditions, mitochondria generate small amounts of ROS as byproducts of oxidative phosphorylation. However, when the electron transport chain becomes dysfunctional, particularly at complexes I and III, excessive electron leakage occurs, leading to ROS overproduction. The resulting oxidative burden promotes lipid peroxidation, protein oxidation, and DNA damage, ultimately impairing cellular function and viability.

Among retinal cells, photoreceptors are especially vulnerable to oxidative damage because of their exceptionally high metabolic activity and the abundance of polyunsaturated fatty acids within their outer segments, which are highly susceptible to peroxidation [14]. This vulnerability is further enhanced by the continuous exposure of the retina to visible light, creating an environment that favors photo-oxidative stress.

Oxidative stress also affects the RPE, a tissue essential for photoreceptor maintenance and retinal homeostasis. Excessive ROS impair RPE phagocytic function and promote the accumulation of lipofuscin and toxic byproducts such as A2E. These compounds further compromise mitochondrial function, leading to additional ROS generation and perpetuating cellular damage [15].

At the molecular level, oxidative stress disrupts several redox-sensitive signaling pathways involved in cellular defense and survival. In particular, alterations of the Nrf2 antioxidant response and activation of NF-κB-dependent inflammatory signaling reduce the ability of retinal cells to cope with environmental and metabolic stressors. Consequently, mitochondrial dysfunction and oxidative stress become tightly interconnected, creating a vicious cycle in which mitochondrial damage enhances ROS production, while ROS further impair mitochondrial integrity and function. This self-perpetuating process is thought to play a major role in disease progression and photoreceptor loss [16].

## 4. Mitophagy and Mitochondrial Quality Control

The maintenance of mitochondrial integrity depends on efficient quality-control mechanisms, among which mitophagy plays a fundamental role. This process allows the selective elimination of damaged or dysfunctional mitochondria, thereby preserving cellular homeostasis and preventing the accumulation of potentially harmful organelles. In the retina, where energy demand is exceptionally high, efficient mitophagy is particularly important for maintaining neuronal survival and function.

The best-characterized pathway regulating mitophagy is the PINK1/Parkin system. Following mitochondrial depolarization, PINK1 accumulates on the outer mitochondrial membrane and recruits the E3 ubiquitin ligase Parkin, which labels damaged mitochondria for autophagic degradation [17]. Through this mechanism, cells can remove dysfunctional mitochondria before they trigger excessive oxidative stress or apoptotic signaling.

When mitophagy becomes impaired, damaged mitochondria accumulate within retinal cells, resulting in increased ROS production, bioenergetic failure, and activation of cell death pathways. Experimental studies have linked defective mitophagy to progressive photoreceptor degeneration and retinal pigment epithelium dysfunction, suggesting that alterations in mitochondrial quality control may contribute directly to disease progression.

Mitochondrial homeostasis is also regulated by the dynamic balance between fission and fusion processes. These events, controlled by proteins such as Drp1, Mfn1, and Mfn2, are essential for maintaining mitochondrial morphology, distribution, and function. Disturbances in this balance can lead to excessive mitochondrial fragmentation and reduced metabolic efficiency. While increased fission has been associated with heightened susceptibility to apoptosis, impaired fusion limits the ability of mitochondria to repair damage and maintain functional competence [12].

Importantly, several experimental studies have shown that enhancing mitophagy or restoring physiological mitochondrial dynamics can reduce retinal damage and improve cell survival. These findings have generated growing interest in mitochondrial quality-control pathways as potential therapeutic targets for retinal degenerative diseases [18].

## 5. Regulated Cell Death Pathways in Retinal Degeneration

In recent years, growing attention has been directed toward the role of regulated cell death pathways in retinal degeneration. While oxidative stress and mitochondrial dysfunction are widely recognized as major contributors to retinal injury, accumulating evidence suggests that they ultimately converge on multiple cell death programs that actively drive photoreceptor and RPE loss. Rather than representing independent processes, apoptosis, ferroptosis, necroptosis, and pyroptosis appear to be closely interconnected and influenced by mitochondrial homeostasis [19,20,21,22].

Among these mechanisms, ferroptosis has emerged as a particularly intriguing pathway. Characterized by iron-dependent lipid peroxidation and overwhelming oxidative damage, ferroptosis has been implicated in both age-related macular degeneration (AMD) and inherited retinal disorders. Experimental studies have demonstrated that impaired mitochondrial metabolism can enhance susceptibility to ferroptotic cell death through disruption of cellular redox balance and depletion of antioxidant defenses [19,20,21]. These findings have stimulated interest in ferroptosis inhibitors as potential neuroprotective strategies for retinal disease.

Inflammatory forms of regulated cell death have also attracted increasing attention. Pyroptosis, mediated by inflammasome activation and caspase-dependent inflammatory signaling, has been observed in retinal pigment epithelium cells exposed to chronic oxidative stress. Activation of the NLRP3 inflammasome appears to contribute not only to cell death but also to the establishment of a persistent inflammatory microenvironment that may accelerate disease progression [23,24].

Similarly, necroptosis has been implicated in photoreceptor degeneration in several experimental models. Unlike apoptosis, necroptosis is characterized by membrane disruption and the release of pro-inflammatory mediators, thereby amplifying tissue damage. Evidence suggests that mitochondrial dysfunction and excessive ROS production may facilitate activation of RIPK-dependent necroptotic pathways, further linking metabolic stress to retinal cell loss [25,26].

Mitochondrial apoptosis remains one of the best-characterized mechanisms of retinal degeneration. Disruption of mitochondrial membrane integrity, cytochrome c release, and activation of downstream caspases have been documented in both AMD and inherited retinal dystrophies. Importantly, oxidative stress and mitochondrial DNA damage appear to sensitize retinal cells to apoptotic signaling, highlighting the close relationship between mitochondrial dysfunction and programmed cell death [27,28].

Taken together, these observations suggest that mitochondrial dysfunction contributes to retinal degeneration not only through impaired energy production but also through activation of multiple, interconnected cell death pathways. A better understanding of the relative contribution of these mechanisms across different retinal diseases may help identify novel therapeutic targets and improve the development of disease-modifying interventions.

## 6. Inflammation and Mitochondrial Crosstalk

Mitochondrial dysfunction and inflammation are increasingly viewed as interconnected components of the pathogenic process underlying retinal degeneration. Beyond their role in cellular metabolism, mitochondria actively participate in immune signaling. When damaged, they release mtDNA, cardiolipin, and other damage-associated molecular patterns (DAMPs), which can be recognized by innate immune receptors and trigger inflammatory responses, including activation of the NLRP3 inflammasome [29].

Activation of these pathways promotes microglial recruitment and stimulates the release of pro-inflammatory cytokines such as IL-1β, IL-6, and TNF-α. Although acute inflammatory responses may initially serve protective functions, persistent activation of retinal microglia contributes to a chronic inflammatory environment that exacerbates neuronal injury and accelerates photoreceptor degeneration.

This inflammatory component is particularly relevant in AMD, where complement system dysregulation has emerged as a major pathogenic mechanism. Genetic studies have identified variants in complement-related genes, including CFH, as important determinants of disease susceptibility and progression, highlighting the complex interaction between innate immunity and retinal degeneration [30].

Oxidative stress further strengthens this connection by activating inflammatory signaling pathways, including NF-κB and other stress-responsive transcription factors. In turn, inflammatory mediators impair mitochondrial function, promote ROS production, and increase cellular vulnerability. As a result, mitochondrial dysfunction, oxidative stress, and inflammation become locked in a self-reinforcing pathogenic network that drives progressive retinal damage [31].

**Figure 1:**
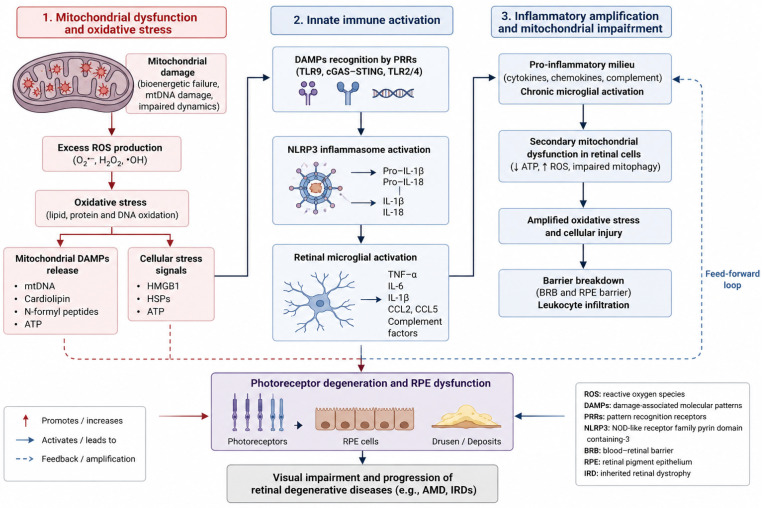
Interplay between mitochondrial dysfunction, oxidative stress, and inflammation in retinal degeneration. Mitochondrial damage leads to excessive production of reactive oxygen species (ROS) and the release of mitochondrial-derived danger-associated molecular patterns (DAMPs), including cell-free mitochondrial DNA (mtDNA) and cardiolipin. These signals activate retinal microglia and inflammasome pathways, promoting the release of pro-inflammatory mediators and amplifying local inflammatory responses. In parallel, oxidative stress further impairs mitochondrial function, resulting in additional ROS generation and accumulation of mitochondrial damage. The reciprocal interaction between mitochondrial dysfunction and inflammation establishes a self-sustaining pathogenic cycle that contributes to progressive retinal pigment epithelium (RPE) dysfunction, photoreceptor degeneration, and disease progression.

The complex bidirectional relationship between mitochondrial dysfunction and inflammation, mediated by ROS and mitochondrial-derived DAMPs, is illustrated schematically in Figure 1.

## 7. Disease-Specific Considerations in Retinal Degeneration

Although mitochondrial dysfunction and oxidative stress are shared mechanisms across several retinal degenerative diseases, their roles are not identical in all conditions. Retinal degeneration encompasses a heterogeneous group of disorders that differ in genetic background, primary cellular targets, disease progression, and therapeutic opportunities. Consequently, mitochondrial impairment should be considered a common pathogenic pathway whose contribution may vary according to the specific disease context [2,31,32].

In AMD, mitochondrial dysfunction primarily affects RPE cells, which play a critical role in maintaining photoreceptor homeostasis. Age-dependent accumulation of mitochondrial DNA damage, reduced mitochondrial biogenesis, impaired oxidative phosphorylation, and chronic oxidative stress contribute to progressive RPE dysfunction [15,31,33]. In addition, complement dysregulation and chronic para-inflammatory responses further exacerbate mitochondrial injury, creating a permissive environment for drusen formation, geographic atrophy, and photoreceptor degeneration [30,34].

In inherited retinal dystrophies, including retinitis pigmentosa (RP), the initiating event is generally a pathogenic mutation affecting photoreceptor structure or function. However, increasing evidence suggests that mitochondrial dysfunction may act as a secondary driver of disease progression. Reduced ATP production, impaired mitochondrial dynamics, and increased oxidative stress have been documented in several RP models, where they appear to accelerate photoreceptor cell death independently of the primary mutation [11,35,36].

Similarly, Stargardt disease and other inherited macular dystrophies exhibit distinct pathogenic mechanisms involving the accumulation of toxic bisretinoids, lipofuscin deposition, and retinal pigment epithelium dysfunction. These processes increase oxidative burden and mitochondrial stress, thereby contributing to retinal degeneration through mechanisms that partially overlap with those observed in AMD [37,38].

Taken together, these observations suggest that mitochondrial dysfunction should not be regarded as a uniform pathogenic process across retinal diseases. Rather, it represents a common biological pathway whose timing, relative contribution, and therapeutic relevance differ among disease entities. Future therapeutic strategies will likely require disease-specific approaches that integrate genetic background, disease stage, and mitochondrial status to maximize clinical efficacy [32,39].

## 8. Biomarkers of Mitochondrial Dysfunction

The identification of reliable biomarkers is a critical step toward earlier diagnosis, improved disease monitoring, and the development of personalized therapeutic strategies for retinal degeneration. Because mitochondrial dysfunction often precedes overt structural damage, biomarkers capable of detecting these early alterations may provide valuable insight into disease activity and progression.

Among the most promising molecular candidates is circulating mtDNA. Released from damaged or stressed cells, cell-free mtDNA can be detected in peripheral blood and has emerged as a potential indicator of mitochondrial injury and cellular stress [40]. As a minimally invasive liquid biopsy approach, circulating mtDNA offers the possibility of monitoring mitochondrial dysfunction dynamically over time, making it an attractive tool for both clinical practice and research.

Biochemical markers of oxidative stress may provide complementary information. Malondialdehyde (MDA), a well-established product of lipid peroxidation, has been widely used as an indicator of oxidative damage and disease burden [41]. Similarly, increased levels of reactive oxygen species and other oxidation-related metabolites have been associated with retinal cellular injury and may reflect disease severity or progression.

Advances in retinal imaging have also expanded the range of available biomarkers. Optical coherence tomography (OCT), now a cornerstone of retinal diagnostics, can provide indirect information about mitochondrial health by assessing the integrity of photoreceptors and RPE [42]. Quantitative OCT parameters, such as alterations in the ellipsoid zone and outer retinal thickness, may serve as sensitive indicators of disease progression and therapeutic response. These imaging biomarkers are particularly valuable because they allow repeated, non-invasive evaluation of retinal structure over time.

Beyond traditional molecular and imaging markers, high-throughput omics technologies are opening new avenues for biomarker discovery. Approaches such as metabolomics and transcriptomics enable the comprehensive characterization of metabolic pathways and gene expression profiles associated with mitochondrial dysfunction. By capturing disease-related molecular signatures, these techniques may facilitate the identification of novel biomarkers and uncover previously unrecognized therapeutic targets.

Taken together, the integration of molecular, imaging, and omics-based biomarkers offers a more comprehensive view of disease biology than any single marker alone. Such multimodal approaches have the potential to improve patient stratification, enable earlier detection of retinal degeneration, and support the development of precision medicine strategies tailored to individual disease characteristics.

Recent studies have identified several additional biomarkers that may improve the assessment of mitochondrial dysfunction in retinal diseases. Markers of oxidative DNA damage, particularly 8-hydroxy-2′-deoxyguanosine (8-OHdG), have been associated with increased oxidative stress and disease severity in AMD and inherited retinal disorders [43,44]. Functional measures of mitochondrial respiration, ATP production, and oxygen consumption rates are increasingly used in experimental studies to evaluate mitochondrial bioenergetics [45].

Extracellular vesicles containing mitochondrial proteins, mitochondrial DNA fragments, and oxidative stress-related molecules have recently emerged as promising liquid biopsy candidates for monitoring retinal disease progression [46]. Advances in retinal imaging, including OCT angiography, adaptive optics imaging, fluorescence lifetime imaging ophthalmoscopy, and quantitative analysis of ellipsoid zone integrity, may further improve the non-invasive assessment of mitochondrial dysfunction and photoreceptor health [47,48,49].

Future biomarker development will likely rely on the integration of molecular, imaging, and multi-omics approaches to provide a comprehensive assessment of disease activity and therapeutic response.

The most relevant biomarkers of mitochondrial dysfunction in retinal degeneration are summarized in Table 2.

## 9. Therapeutic Strategies Targeting Mitochondrial Dysfunction

The growing recognition of mitochondrial dysfunction as a key contributor to retinal degeneration has stimulated considerable interest in the development of mitochondria-targeted therapeutic strategies. Although no therapy specifically designed to restore mitochondrial function has yet been approved for retinal degenerative diseases, several approaches have shown encouraging results in preclinical studies and are currently being explored as potential disease-modifying interventions [31,32].

### 9.1. Antioxidant Therapies

Because excessive production of ROS is a central feature of mitochondrial dysfunction, antioxidant therapies represent one of the most extensively investigated approaches. Conventional antioxidants, including vitamins C and E, lutein, zeaxanthin, and omega-3 fatty acids, have been evaluated primarily in AMD. The AREDS and AREDS2 studies demonstrated that nutritional supplementation may slow progression in selected patients with intermediate AMD; however, these treatments do not directly target mitochondrial dysfunction, and their overall effects remain modest [50].

More recently, mitochondria-targeted antioxidants have been developed to overcome the limitations of conventional antioxidant therapies. Among these, MitoQ, a coenzyme Q10 derivative selectively accumulated within mitochondria, has shown the ability to reduce oxidative damage, improve mitochondrial function, and preserve retinal structure in experimental models [51]. Similarly, SkQ1 and other mitochondria-targeted compounds have demonstrated protective effects against oxidative stress-induced retinal injury, although clinical evidence remains limited [52].

### 9.2. Modulation of Mitochondrial Biogenesis and Quality Control

Strategies aimed at enhancing mitochondrial biogenesis have emerged as another promising therapeutic avenue. Particular attention has focused on peroxisome proliferator-activated receptor gamma coactivator-1 alpha (PGC-1α), a master regulator of mitochondrial metabolism and oxidative phosphorylation. Experimental studies have shown that activation of PGC-1α signaling can improve mitochondrial function, increase cellular resistance to oxidative stress, and promote photoreceptor survival [10,34].

Restoration of mitochondrial quality-control mechanisms has also attracted increasing interest. Impaired mitophagy contributes to the accumulation of dysfunctional mitochondria and the amplification of oxidative stress. Consequently, pharmacological interventions targeting the PINK1/Parkin pathway or modulating mitochondrial fission and fusion processes have shown neuroprotective effects in several experimental models [17,18,53]. Although these approaches remain largely preclinical, they provide compelling evidence that mitochondrial turnover represents a potentially druggable target in retinal degeneration.

### 9.3. Metabolic Interventions

Mitochondrial function is closely linked to cellular metabolism, making metabolic therapies attractive candidates for retinal neuroprotection. Nicotinamide adenine dinucleotide (NAD^+^) plays a fundamental role in mitochondrial energy production and cellular stress responses. Experimental studies have demonstrated that NAD^+^ supplementation, as well as administration of its precursors such as nicotinamide riboside and nicotinamide mononucleotide, can improve mitochondrial function, reduce oxidative stress, and delay retinal degeneration in animal models [54,55].

Additional metabolic approaches include activation of sirtuins and AMP-activated protein kinase (AMPK), both of which regulate mitochondrial biogenesis, autophagy, and cellular energy homeostasis. Although preliminary findings are encouraging, further investigation is required to determine their long-term safety and efficacy in retinal diseases [56].

### 9.4. Translational Challenges and Future Perspectives

Several innovative therapeutic strategies are currently being explored beyond conventional antioxidant supplementation. Gene-based therapies aimed at restoring mitochondrial homeostasis represent a rapidly evolving field. Experimental approaches targeting mitochondrial biogenesis regulators, antioxidant defense pathways, and mitochondrial DNA maintenance have demonstrated encouraging results in preclinical studies [57,58]. Mitochondrial transplantation has recently emerged as a novel therapeutic concept. Transfer of healthy mitochondria into damaged retinal tissues has shown neuroprotective effects in experimental models, although important challenges regarding delivery, long-term survival, and safety remain unresolved [59,60]. Stem cell-based therapies may also provide indirect mitochondrial support through paracrine neuroprotective mechanisms, modulation of inflammation, and transfer of healthy mitochondria to injured retinal cells [61]. Additional strategies include pharmacological activation of the Nrf2 antioxidant pathway, modulation of mitophagy, and inhibition of ferroptosis. Nrf2 activators have demonstrated the ability to reduce oxidative damage and improve retinal cell survival in several experimental models, while ferroptosis inhibitors have shown promising neuroprotective effects in photoreceptor degeneration [62,63,64,65].

Despite these advances, most mitochondrial-targeted therapies remain at the preclinical stage, highlighting the need for rigorous clinical trials to establish efficacy, safety, optimal timing of intervention, and patient selection criteria. Despite the growing number of experimental interventions, the translation of mitochondrial-targeted therapies into clinical practice remains challenging. Most available evidence derives from cell culture and animal studies, while robust clinical trials in patients with retinal degenerative diseases are still lacking. Furthermore, significant barriers remain regarding drug delivery, as many compounds exhibit limited penetration across the blood–retinal barrier or insufficient accumulation within target retinal cells [66].

Another important consideration is the timing of intervention. Since mitochondrial dysfunction may occur early during disease progression, therapies targeting mitochondrial pathways are likely to be most effective before irreversible photoreceptor loss has occurred [11]. This highlights the need for sensitive biomarkers capable of identifying patients at risk and monitoring treatment response.

Finally, the marked heterogeneity of retinal degenerative diseases suggests that therapeutic strategies may need to be tailored according to the underlying disease mechanism, genetic background, and stage of progression. Future advances in molecular diagnostics, imaging biomarkers, and precision medicine approaches may facilitate the identification of patient subgroups most likely to benefit from mitochondria-targeted interventions [32,39].

Taken together, current evidence supports mitochondrial pathways as promising therapeutic targets for retinal degeneration. However, substantial work remains to bridge the gap between experimental findings and clinical application, and well-designed translational studies will be essential to determine whether these approaches can meaningfully alter the course of retinal disease.

**Figure 2:**
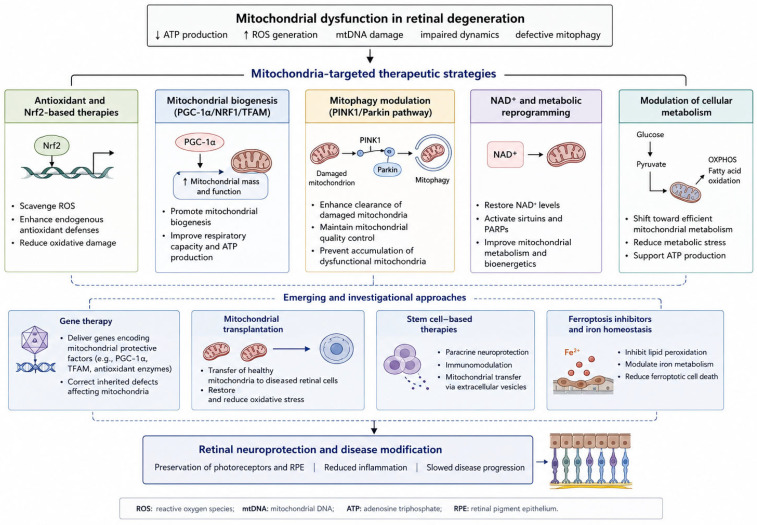
Current and emerging mitochondria-targeted therapeutic strategies for retinal degeneration. Mitochondrial dysfunction represents a promising therapeutic target in retinal degenerative diseases. Current approaches aim to reduce oxidative stress through antioxidant and Nrf2-mediated pathways, enhance mitochondrial biogenesis via activation of PGC-1α-dependent signaling, restore mitochondrial quality control through modulation of mitophagy, and improve cellular metabolism through NAD^+^ supplementation and metabolic reprogramming. Emerging strategies, including gene therapy, mitochondrial transplantation, stem cell-based interventions, and ferroptosis inhibitors, seek to directly address the mechanisms underlying mitochondrial damage and retinal cell loss. Collectively, these approaches aim to preserve photoreceptor and retinal pigment epithelium function, delay disease progression, and promote retinal neuroprotection.

The main mitochondrial-targeted therapeutic strategies and their mechanisms of action are illustrated in Figure 2. Despite promising preclinical results, the translation of mitochondrial-targeted therapies into clinical practice remains limited, highlighting the need for well-designed randomized controlled trials.

## 10. Discussion

**Figure 3:**
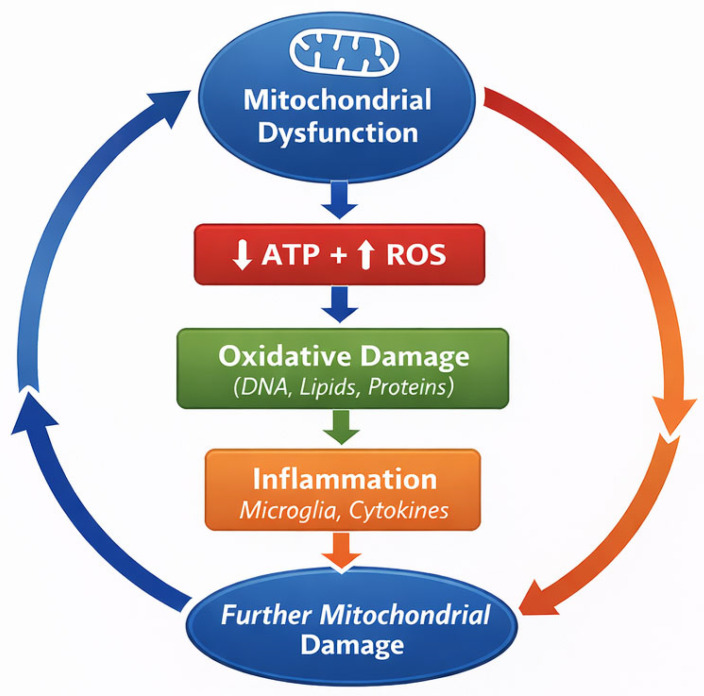
Integrated pathogenic model linking mitochondrial dysfunction, oxidative stress, inflammation, and retinal cell death. Mitochondrial dysfunction results in impaired oxidative phosphorylation, reduced ATP production, and increased generation of reactive oxygen species (ROS). Excessive ROS promote mitochondrial DNA damage, lipid peroxidation, and protein oxidation, further aggravating mitochondrial impairment. Damaged mitochondria release pro-inflammatory signals that activate microglia and inflammasome pathways, amplifying retinal inflammation. These events converge on multiple regulated cell death pathways, including apoptosis, ferroptosis, pyroptosis, and necroptosis, ultimately leading to photoreceptor and retinal pigment epithelium degeneration. The figure illustrates the interconnected nature of these mechanisms and highlights potential points for therapeutic intervention.

This review provides a comprehensive synthesis of current evidence supporting mitochondrial dysfunction and oxidative stress as central and interconnected mechanisms in retinal degeneration. Available evidence suggests that mitochondrial dysfunction may contribute to the early phases of retinal degeneration. However, whether mitochondrial impairment acts as a primary causal driver or a secondary disease modifier remains incompletely understood and likely differs among disease entities. While experimental studies support an important pathogenic role for mitochondrial dysfunction, much of the current evidence remains associative and derives from preclinical models [2,3,11]. The interplay between mitochondrial dysfunction, oxidative stress, and inflammation forms a self-amplifying pathogenic loop (Figure 3).

The evidence reviewed in this article supports the growing recognition that mitochondrial dysfunction and oxidative stress are central contributors to retinal degeneration. Rather than being isolated events, these processes appear to interact through a complex network of molecular pathways that ultimately drive photoreceptor loss and retinal damage. Based on the available data, it is possible to envision a unified pathogenic model in which mitochondrial impairment occurs early in the disease course and triggers a cascade involving oxidative stress, defective mitochondrial quality control, and chronic inflammation, all of which contribute to progressive retinal degeneration.

One of the most important concepts emerging from recent studies is the existence of a self-perpetuating cycle linking mitochondrial dysfunction and oxidative stress. When the electron transport chain becomes impaired, mitochondrial efficiency declines and ROS production increases. These reactive molecules damage mitochondrial DNA, proteins, and membrane lipids, further compromising mitochondrial function and leading to even greater oxidative stress [7,8,13,16]. This vicious cycle may be particularly harmful in retinal tissue, where energy requirements are exceptionally high, and cells are continuously exposed to light-induced oxidative challenges [3,4]. Over time, the accumulation of mitochondrial DNA alterations and oxidative damage may progressively reduce the ability of retinal cells to maintain metabolic homeostasis, contributing to cellular dysfunction and degeneration [8,9].

Another key aspect is the role of mitochondrial quality-control mechanisms. Healthy retinal function depends on the continuous removal of damaged mitochondria and the maintenance of a balanced mitochondrial network. Impaired PINK1/Parkin-mediated mitophagy results in the persistence of dysfunctional mitochondria, increased oxidative stress, and activation of apoptotic pathways [17,18]. At the same time, disturbances in mitochondrial fission and fusion dynamics can promote mitochondrial fragmentation and compromise bioenergetic efficiency [6,12]. Together, these findings highlight the importance of preserving mitochondrial turnover and structural integrity, suggesting that interventions targeting these pathways may offer neuroprotective benefits.

The relationship between mitochondrial dysfunction and inflammation adds another layer of complexity to retinal degeneration. Damaged mitochondria can act as potent sources of inflammatory signals through the release of mitochondrial DNA and other danger-associated molecular patterns (DAMPs), which activate innate immune pathways and inflammasomes [29]. The resulting microglial activation and cytokine release contribute to a chronic inflammatory environment that further damages retinal tissue. In diseases such as age-related macular degeneration, this process is compounded by complement dysregulation, with genetic variants in complement-related genes, including CFH, playing an important role in disease susceptibility and progression [30,31]. Consequently, mitochondrial dysfunction and inflammation appear to reinforce one another, creating a feed-forward mechanism that accelerates retinal damage.

From a therapeutic perspective, these findings provide both opportunities and challenges. Conventional antioxidant therapies have shown some ability to reduce oxidative injury, but their clinical benefits have generally been modest, partly because of limited bioavailability and poor targeting of the primary source of ROS production [50]. In contrast, mitochondria-targeted antioxidants such as MitoQ have generated considerable interest because they selectively accumulate within mitochondria and may more effectively counteract oxidative stress at its origin [67]. Similarly, strategies aimed at stimulating mitochondrial biogenesis through activation of PGC-1α and related pathways have demonstrated encouraging results in experimental studies, improving cellular energy metabolism and resistance to oxidative damage [10].

Additional therapeutic approaches are emerging from the field of metabolic modulation. Interventions based on NAD^+^ supplementation, sirtuin activation, and related metabolic pathways have shown potential for enhancing mitochondrial function and improving cellular resilience [54]. Although these strategies are conceptually attractive and supported by promising preclinical evidence, their clinical applicability remains to be established through rigorous human studies.

An important consideration is the timing of intervention. Multiple studies suggest that mitochondrial abnormalities arise before overt structural degeneration becomes clinically detectable [11]. This observation raises the possibility that therapies targeting mitochondrial pathways may be most effective when administered during the earliest stages of disease, before irreversible photoreceptor loss has occurred. In this context, the development of sensitive and reliable biomarkers capable of detecting early mitochondrial dysfunction becomes particularly important [40,41].

Despite the substantial progress achieved over the past decade, significant challenges remain. Retinal degenerative diseases are highly heterogeneous, with complex interactions between genetic, metabolic, and environmental factors. This heterogeneity complicates both mechanistic studies and therapeutic development. Moreover, differences among experimental models and study methodologies often limit direct comparison across investigations. Perhaps most importantly, much of the current evidence derives from in vitro and animal studies, and successful translation to human disease remains an ongoing challenge.

Future research should focus on integrating molecular, imaging, and multi-omics data to achieve a more comprehensive understanding of disease mechanisms. Combining genomics, transcriptomics, metabolomics, and advanced retinal imaging may facilitate the identification of disease-specific pathways and improve patient stratification. Such approaches could ultimately support the development of personalized therapeutic strategies tailored to individual biological profiles. At the same time, well-designed clinical trials are urgently needed to determine whether mitochondrial-targeted interventions can meaningfully alter disease progression in patients.

Overall, the available evidence strongly suggests that mitochondrial dysfunction and oxidative stress are not merely consequences of retinal degeneration but active contributors to disease development and progression. Their close interaction with inflammatory pathways and genetic susceptibility highlights the multifactorial nature of retinal degenerative disorders. Although considerable work remains before these insights can be fully translated into clinical practice, targeting mitochondrial pathways represents one of the most promising directions for future therapeutic development.

This review has several limitations that should be acknowledged. First, the studies included were heterogeneous in terms of experimental design, disease models, and outcome measures, limiting direct comparisons across investigations. Second, most of the available evidence is derived from preclinical studies, which may not fully reflect the complexity of human retinal disease. Third, publication bias cannot be excluded, as positive findings are more likely to be reported than negative or inconclusive results. Finally, the absence of a quantitative meta-analysis limits the ability to draw definitive conclusions regarding the magnitude of the observed effects.

### Knowledge Gaps and Future Research Priorities

Several important questions remain unanswered. First, the precise causal role of mitochondrial dysfunction in different retinal diseases remains incompletely understood. Whether mitochondrial abnormalities represent primary drivers of degeneration or secondary amplifiers of disease progression may vary according to disease entity and stage.

Second, the translation of promising experimental therapies into clinical practice has proven challenging. Differences between animal models and human disease, limited availability of early biomarkers, and difficulties in retinal drug delivery continue to hinder clinical development.

Third, advances in single-cell transcriptomics, spatial transcriptomics, proteomics, and metabolomics are beginning to reveal previously unrecognized cellular heterogeneity within the degenerating retina. Future studies integrating these approaches may improve patient stratification and facilitate the development of precision medicine strategies.

Finally, well-designed clinical trials focusing on mitochondrial-targeted interventions are urgently needed to determine whether modulation of mitochondrial pathways can meaningfully alter visual outcomes in patients with retinal degeneration.

## 11. Conclusions

Mitochondrial dysfunction and oxidative stress have emerged as fundamental and closely interconnected mechanisms in the pathogenesis of retinal degenerative diseases. Growing evidence suggests that these processes are not simply secondary consequences of retinal injury but may actively contribute to the initiation and progression of degeneration. Through their effects on cellular energy metabolism, reactive oxygen species production, and inflammatory signaling, alterations in mitochondrial homeostasis can profoundly influence retinal survival and function.

Over the past decade, substantial advances have improved our understanding of the molecular pathways linking mitochondrial impairment to retinal degeneration. These discoveries have identified several potential therapeutic targets and have stimulated the development of novel strategies aimed at preserving mitochondrial function. In particular, interventions designed to reduce oxidative stress, enhance mitophagy, restore mitochondrial quality control, or promote mitochondrial biogenesis have shown encouraging results in experimental models, highlighting the therapeutic potential of mitochondria-directed approaches.

Despite these promising developments, translating preclinical findings into effective clinical therapies remains challenging. The complexity and heterogeneity of retinal degenerative diseases, together with the predominance of evidence derived from laboratory and animal studies, continue to limit clinical application. In this context, the identification of robust biomarkers capable of detecting early mitochondrial dysfunction and monitoring disease progression will be essential for improving patient stratification, guiding treatment decisions, and evaluating therapeutic response.

Looking ahead, future research should focus on strengthening the link between experimental discoveries and clinical practice. Well-designed clinical trials, combined with advances in molecular profiling and precision medicine, will be critical for determining the true therapeutic value of mitochondrial-targeted interventions. A more comprehensive understanding of the intricate relationship among mitochondrial dysfunction, oxidative stress, and inflammation may ultimately pave the way for personalized treatment strategies capable of slowing disease progression and preserving vision in patients affected by retinal degeneration.

Ultimately, targeting mitochondrial pathways represents one of the most promising avenues for future retinal neuroprotection. While significant challenges remain, continued progress in this field may lead to innovative therapies that address the underlying mechanisms of disease rather than simply managing its consequences.

## Figures and Tables

**Table 1:** Key mechanisms linking mitochondrial dysfunction to retinal degeneration.

Mechanism	Molecular Features	Cellular Consequences	Evidence
Mitochondrial structural damage	Cristae disruption, swelling, fragmentation	Impaired oxidative phosphorylation	[5,6]
Electron transport chain dysfunction	Reduced ATP production, electron leakage	Increased ROS generation	[7]
mtDNA damage	Mutations, deletions	Reduced mitochondrial efficiency	[8,9]
Impaired mitochondrial biogenesis	PGC-1α dysregulation	Reduced compensatory capacity	[10]
Altered mitochondrial dynamics	Excessive fission, reduced fusion	Increased apoptosis susceptibility	[12]

**Table 2:** Biomarkers of mitochondrial dysfunction and oxidative stress in retinal degeneration.

Biomarker	Category	Biological Significance	Clinical Relevance
Circulating cell-free mtDNA	Molecular (liquid biopsy)	Released from damaged mitochondria; reflects mitochondrial membrane disruption and cellular stress	Non-invasive marker of mitochondrial damage and disease activity
Malondialdehyde (MDA)	Lipid peroxidation marker	End-product of polyunsaturated fatty acid oxidation induced by ROS	Indicator of oxidative damage severity and disease progression
Reactive oxygen species (ROS)	Cellular oxidative marker	Includes superoxide anion, hydrogen peroxide, and hydroxyl radicals generated by mitochondrial dysfunction	Reflects intracellular oxidative burden (mainly experimental use)
Lipofuscin/A2E	RPE-specific metabolic marker	Accumulation of bisretinoids from incomplete phagocytosis of photoreceptor outer segments	Associated with RPE dysfunction and AMD progression
Optical coherence tomography (OCT) biomarkers	Imaging	Structural alterations of photoreceptors (ellipsoid zone) and RPE integrity	Non-invasive monitoring of retinal degeneration and therapeutic response

## Data Availability

No new data were created or analyzed in this study. Data sharing is not applicable to this article.

## Abbreviations

The following abbreviations are used in this manuscript:

AMD	Age-related macular degeneration
ATP	Adenosine triphosphate
CFH	Complement factor H
DAMPs	Damage-associated molecular patterns
IL	Interleukin
MDA	Malondialdehyde
mtDNA	Mitochondrial DNA
NAD^+^	Nicotinamide adenine dinucleotide
NF-κB	Nuclear factor kappa B
NLRP3	NOD-like receptor family pyrin domain-containing 3
OCT	Optical coherence tomography
PGC-1α	Peroxisome proliferator-activated receptor gamma coactivator 1-alpha
PINK1	PTEN-induced kinase 1
RPE	Retinal pigment epithelium
ROS	Reactive oxygen species
TNF-α	Tumor necrosis factor alpha
